# Supplementary figures and images for: Evolution of a novel chimeric maltotriose transporter in Saccharomyces eubayanus from parent proteins unable to perform this function
Source: PLoS Genet. 2019 Apr 4;15(4):e1007786. doi: 10.1371/journal.pgen.1007786 (PMC6448821; doi:10.1371/journal.pgen.1007786)

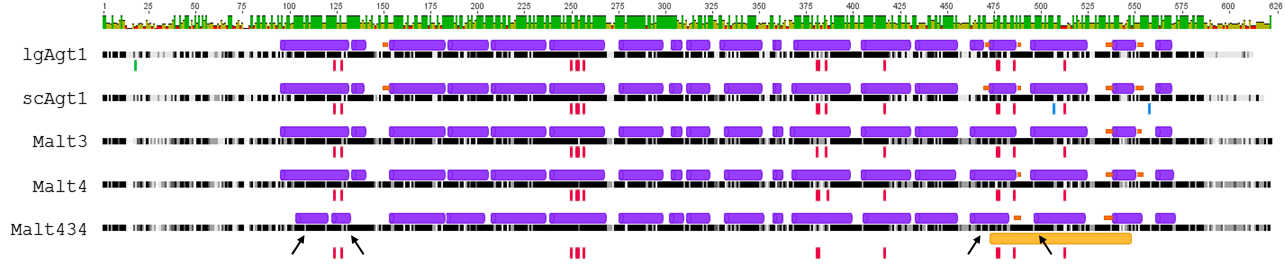

Supplement: S1 Fig — Protein structural alignment between Malt3, Malt4, Malt434, scAgt1, and lgAgt1. The purple blocks represent predicted alpha helices, and the orange lines represent predicted beta strands. Red ticks mark predicted maltose-binding sites. Blue ticks mark residues found to be important for maltotriose transport by Smit et al. 2008. A green tick marks the location of the single non-synonymous substitution between lgAGT1 and tbAGT1. Arrows point to alpha helices in Malt434 whose predicted sizes are reduced compared to other transporters in the alignment. (TIF) [file pgen.1007786.s001.tif]
